# Comparing the un-comparable: Olyset Plus and Olyset, different malaria impact

**DOI:** 10.1186/s12936-018-2596-7

**Published:** 2018-11-30

**Authors:** Ole Skovmand

**Affiliations:** Intelligent Insect Control, Montpellier, France

**Keywords:** Bed net, Insecticide resistance, Olyset Plus, Permethrin, WHO

## Abstract

**Background:**

In a recent article in *The Lancet*, Protopopoff et al. stated that insecticide resistance must be tackled and concluded that adding the insecticide synergist piperonyl butoxide (PBO) to a bed net with a pyrethroid as principal insecticide might be a part of the response.

**Main text:**

The study in Tanzania compares malaria prevalence between users of two different nets with the principal insecticide permethrin: Olyset and Olyset Plus (Olyset+), the latter also holding the synergist molecule PBO, the first not. The article is based on randomized cluster trial of very high quality, but Olyset+ exposes much more permethrin at the surface so the higher efficacy may not be because of the added PBO.

**Conclusion:**

Data published by the World Health Organization (WHO) when evaluating Olyset+ as well of the study of Protopopoff et al. showed that much more permethrin is available on the surface of Olyset+ than on the surface of Olyset and the relatively small and rapidly dwindling dosage of PBO may have nothing to do with the superior effect of Olyset+. The WHO should not change politics for “PBO nets” based on this study alone.

## Background

Pyrethroid resistance has been reported for most of the major malaria vectors in sub-Sahelian Africa and this has often been used as an argument to explain reduced effect of long-lasting insecticidal bed nets (LLINs). Several bed net producers have responded to this by adding the synergist piperonyl butoxide (PBO) to a net with a pyrethroid thus targeting one of the resistance mechanisms identified, the P450 cytochrome or mixed function oxidase that can metabolize pyrethroids. One such net is Olyset Plus (Olyset+) that has added PBO and has been compared in a randomized, multi-cluster study in Tanzania to Olyset that only has permethrin [1].

The World Health Organization (WHO) has until recently recommended PBO nets to be placed among the same product class as with pyrethroid only LLINs, cautiously underlining that there was no proof of the additive effect of PBO in areas of resistance. This caution was further enforced when a multi-country study showed that pyrethroid-only nets worked as well in countries with high pyrethroid resistance as in countries with no pyrethroid resistance, and that coverage and use rate drove the efficacy, not resistance problems [2].

However, WHO has now recommended that the PBO nets may be in a new product class following evidence from the Tanzanian study comparing Olyset+ (permethrin+ PBO) to Olyset (permethrin only) [3]. The present article refers published data to show that this decision is not well founded and add technical information to explain why the nets are different beside the content of PBO.

## Main text

First, it should be remarked that the amount of synergist added in the Olyset+ is relatively small. In most products combining the synergist with a pyrethroid or pyrethrins (like in aerosol cans), 3–10 more PBO is used than that of the pyrethroid, here the level is the half.

However, the major issue is that the release rate of permethrin is different in the yarns of the two nets. This was reported in the WHO evaluation reports of 2012 on Olyset+ [4]. Initially, the WHO reference laboratory for studies on bed nets at IRD in Montpellier determined the so-called regeneration time, the time it takes a net to provide a stable level of mortality after three washings by following it daily with bioassays with a fully susceptible mosquito strain [5]. Olyset started with a mortality rate of 63% before any washing versus 100% for Olyset+ that thus worked better even on susceptible mosquitoes. Regeneration time was not reached for Olyset net in this study after 7 days. For Olyset+, a stable level was reached after 2–7 days at 85% mortality, thus higher mortality than the unwashed Olyset. WHO chose the 2 days for wash intervals in all succeeding tests and not 5, the mid-value. Both nets had the target dosage of 20 g permethrin per kg net at start. After the three washes, Olyset had 19.8 g permethrin/kg and Olyset+ had 16, showing that 20 times more permethrin was washed off from Olyset+ and thus was available on the yarn surface. The report concludes: “*The differential permethrin AI load between Olyset Plus and Olyset Net was due to the bleed rate of the ingredient into the net surface, which is higher in Olyset Plus than in Olyset Net*”. The additional mosquito effect of PBO can, therefore, not be known by comparing the two when the surface dosages of permethrin are so different.

The chemical analysis also included the PBO. Whereas Olyset+ had 64% permethrin left after 20 washes, it just had 44% of the PBO. The ratio PBO to permethrin thus decreased from 0.5:1 to 0.3:1. Since PBO migrated better than the permethrin this difference will be bigger with longer time between washes as in real life use as shown in the study of Protopopoff et al. [1].

In this study, Olyset had 21.4, 21.4 and 16.7 g permethrin/kg net at 0, 12 and 21 months, for Olyset+ the numbers were 20.9, 14.7 and 12.2 and the PBO declined from 9.5 to 1.6 g/kg net, the PBO to permethrin ratio thus declined to 0.16:1. Since losses are correlated to surface concentrations and not in-yarn concentrations, very little permethrin was available in the first year for users of Olyset. The article quoted another article from 2004 [6] that the authors say showed that a higher dosage of permethrin does not increase impact on free flying resistant mosquitoes. This is a misquote. The article concludes the opposite and the resistance mechanism it deals with is *kdr* resistance that is not concerned by PBO.

For the reader to understand, it must be known that these nets are made with a technology of incorporation, meaning that the active ingredients, the insecticide and the synergist, are mixed into the basic polymer of the yarn. The active ingredients can then migrate from the matrix of the yarn to the surface. The dosage of insecticide inside the yarn has no impact on mosquitoes, only the surface concentration has. The speed of the migration depends on the matrix, especially the crystalline density of the matrix. The denser, the slower the migration and the less on the surface, since the surface concentration is a result of this migration, evaporation from the surface and washing and rubbing off. Therefore, even Olyset and Olyset+ has the same amount of permethrin incorporated from start, the concentration at the surface of the yarns caused by different “bleeding rates” of the insecticide is very different. How are these differences obtained? These nets are made of polyethylene. Most of the yarns consist of High Density Polyethylene (HDPE) which provides a strong yarn but allows for very little insecticide migration. Contrary to that, Low Density Polyethylene (LDPE and LLDPE) is much less crystalline and thus allows for a higher migration of additives, which is the reason that e.g. EU has different rules for polyethylene products in contact with food for HDPE than for LDPE/LLDPE. In line with this, the producer of Olyset have issued several patents applications on the best mix of high and low-density polyethylene to have an optimal migration of permethrin and PBO [7, 8]. Olyset+ is based on these patents, but Olyset is outside this range with too much HDPE and, therefore, little surface permethrin.

This does not mean that so-called PBO nets will not work better than so-called standard nets that only holds a pyrethroid. It just means that it has not been proven in this study opposite to what claimed and that institutions like WHO should take a better look on their own data before claiming a policy shift.

## Conclusion

The published study [1] comparing Olyset+ to Olyset showed that Olyset+ has larger impact on malaria than Olyset using the best methodology for comparing two products in a randomized, multi-cluster study. However, the methodology does not reveal why Olyset+ is better, but this is reported in the WHO studies published [4] when the net was recommended: Olyset+ exposure much more permethrin to mosquitoes than Olyset does and the difference between the effect cannot be attributed to the low and rapidly dwindling amount of PBO. The WHO must find a better study to change policy for PBO nets.
